# Supplementary material for: An in-plane magnetic chiral dichroism approach for measurement of intrinsic magnetic signals using transmitted electrons
Source: Nat Commun. 2017 May 15;8:15348. doi: 10.1038/ncomms15348 (PMC5440662; doi:10.1038/ncomms15348)
Supplement: Supplementary Information — Supplementary Figures and Supplementary Notes. [file ncomms15348-s1.pdf]

Supplementary Note 1

Asymmetry of out-of-plane EMCD signals

We define A as the summation of the coefficient from the upper and lower half diffraction plane for the EMCD signal of z component as follows,

$$A = (a_{\text{upper}} + a_{\text{lower}})/2$$

where a_{upper} and a_{lower} are the relative intensities of the EMCD signal for the z component in the upper and lower quadrants (see Supplementary Figure 1). For smaller values of A, the signal from the z component approaches zero if the detector is placed on the x axis and is almost ten times smaller than the relative intensity of the EMCD signal from the in-plane (y) magnetization, *i.e.*, it is negligible. In TEM mode, only the magnetization of the z component remains and should provide the strongest intensity of the EMCD signal because it has the largest intrinsic signal and dynamical diffraction coefficients. Even in this case, we did not measure any signal by placing the detector on the x axis (see Fig. 4(c) in the main text), confirming the negligible signal from the z component in the in-plane diffraction geometry.

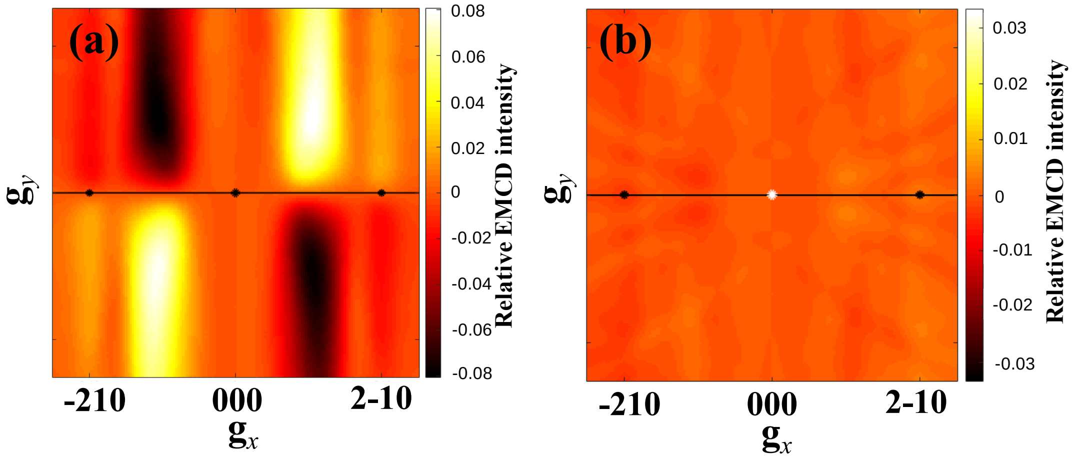

Supplementary Figure 1 | Asymmetry of EMCD signals for *z* component. (a) EMCD signal for the z component under (-210) three-beam case; (b) Summation of the coefficient from the upper and lower half diffraction plane for the EMCD signal of z component (as defined with A in the text).

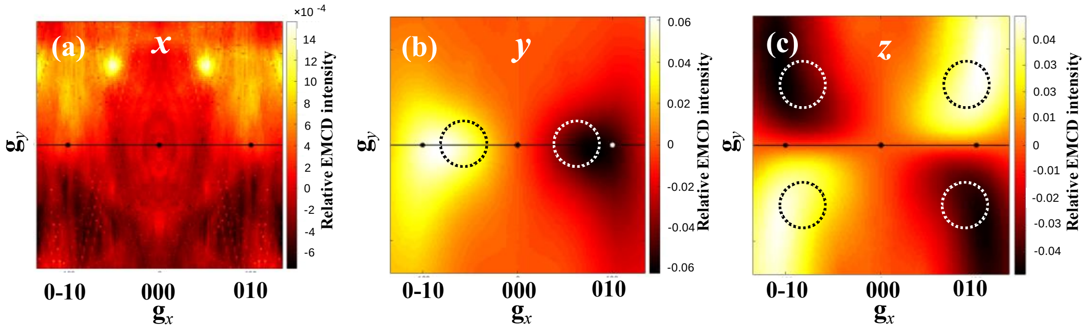

Supplementary Figure 2 | Simulated reciprocal space distributions of the EMCD signal for the *x*, *y* and *z* components in an (010) three-beam geometry for a specimen thickness of 20 nm and an accelerating voltage of 300 kV. For each component, it is assumed that the magnetization is saturated in this direction in the simulations. The black and white spots mark the positions of the transmitted and diffracted beams in the diffraction plane. The indices of the beams are marked along the coordinate axis. The white and black circles mark the detector positions.

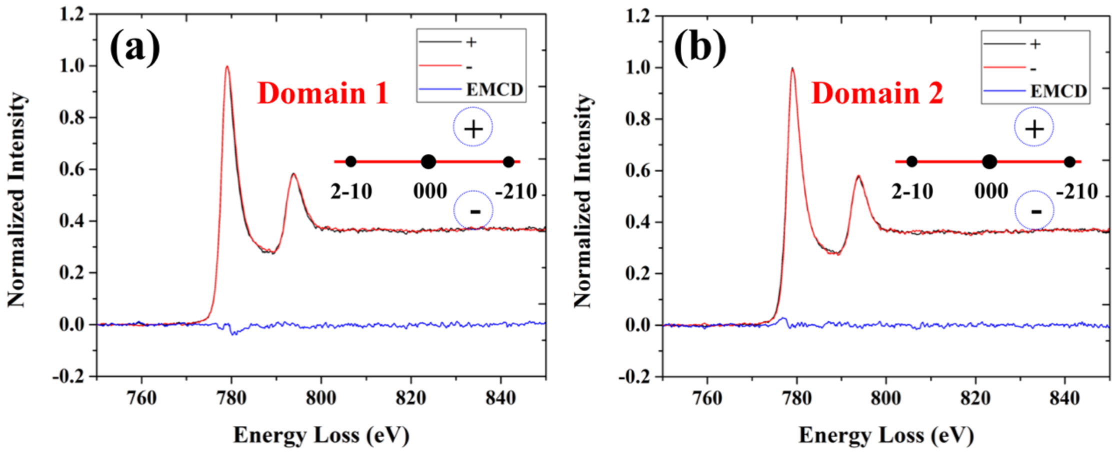

Supplementary Figure 3 | Experimental EMCD signals recorded from Co in out-of-plane diffraction geometries in Lorentz mode. (a, b) show EMCD signals measured in a three beam geometry from domains 1 and 2, respectively, in an out-of-plane diffraction geometry. The schematic diagrams show the diffraction geometry. The blue circles mark the positions of the EELS entrance aperture.

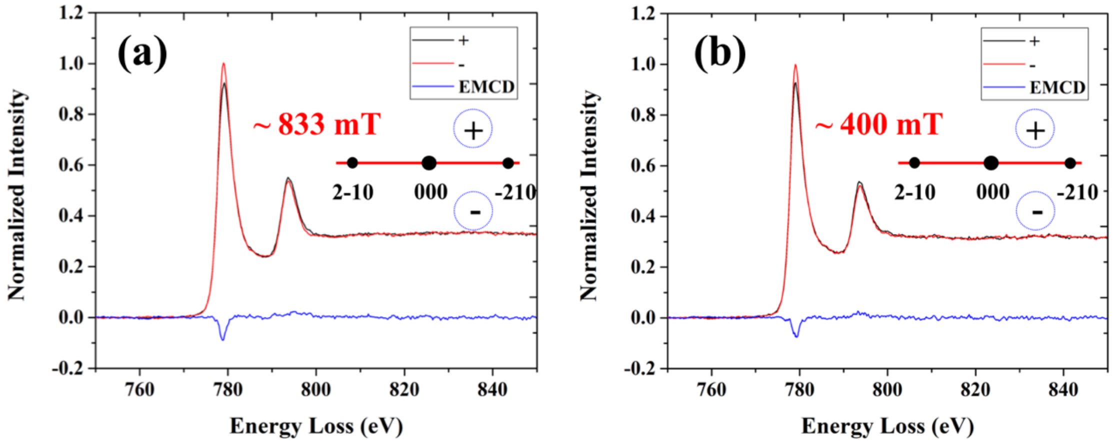

Supplementary Figure 4 | Experimental EMCD signals recorded from Co in an out-of-plane diffraction geometry in Lorentz mode in the presence of an applied magnetic field of (a) ~833 and (b) ~400 mT in the electron beam direction. The schematic diagrams show the diffraction geometry. The blue circles mark the positions of the EELS entrance aperture.

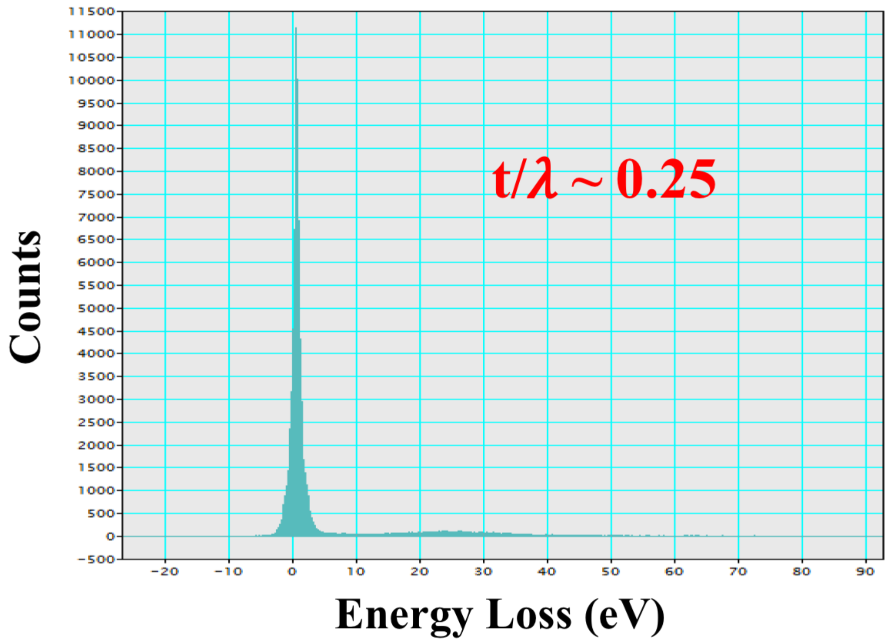

Supplementary Figure 5 | Low-loss EEL spectrum recorded from the Co nanoplate. The relative thickness is 0.25λ, where λ is the effective mean free path for inelastic scattering. λ was estimated to be approximately 80 nm by using an empirical formula for the present experimental conditions. The specimen thickness is therefore inferred to be approximately 20 nm.

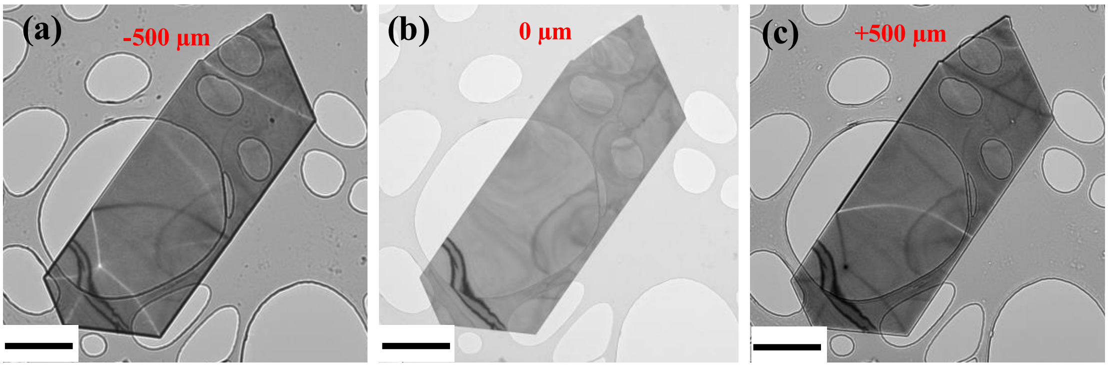

Supplementary Figure 6 | Lorentz TEM images recorded from the Co nanoplate in magnetic-field-free conditions after turning off the conventional microscope objective lens. (a-c) show Fresnel images acquired (a) underfocus, (b) in focus and (c) overfocus. The defocus value Δz used in (a) and (c) was ±500 μm. The magnetic domain structures returned to their original states, which are shown in Fig. 3 in the manuscript. The black scale bar denotes 5 μm.

Supplementary Note 2

Supercell of *hcp* Co for simulations

The supercell for *hcp* Co with an orthogonal system is shown Supplementary Figure 7. The simulations are based on this supercell. The (100) planes in the supercell correspond to (-210) planes in the primary unit cell, while the (010) planes correspond to (010) planes in the primary unit cell.

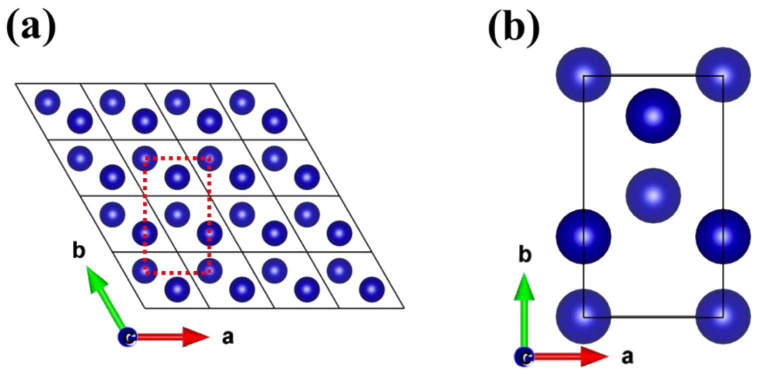

Supplementary Figure 7 | Supercell of *hcp* Co for simulations. (a) Primary cell with 4*4 unit cells projected along the (001) direction. (b) Supercell used for simulations, taken from the red rectangle box in (a).

Supplementary Note 3

Effect of strain on the intensity of the EMCD signal

Small amounts of strain will lead to a slight distortion of the crystal structure and may also further change the magnetic state of the materials. Here, we perform a simulation for *hcp* Co with both -3% compressive strain and 3% tensile strain in the (001) plane, which will lead to a change in lattice parameters. To simplify this, we assume that both the lattice parameter *a* and *b* are changed by -3% or 3%, respectively. The lattice parameter *c* is calculated by assuming that the volume of the unit cell is not changed. The results are shown in Supplementary Figure 8. We find that there is almost negligible difference in the relative intensity of the EMCD signal between the different strain states. It should be noted that the magnetic state is assumed to be same for these three cases. However, if the strain will result in a significant change in the magnetic moment, then it may be possible to detect the obvious change in EMCD signal experimentally.

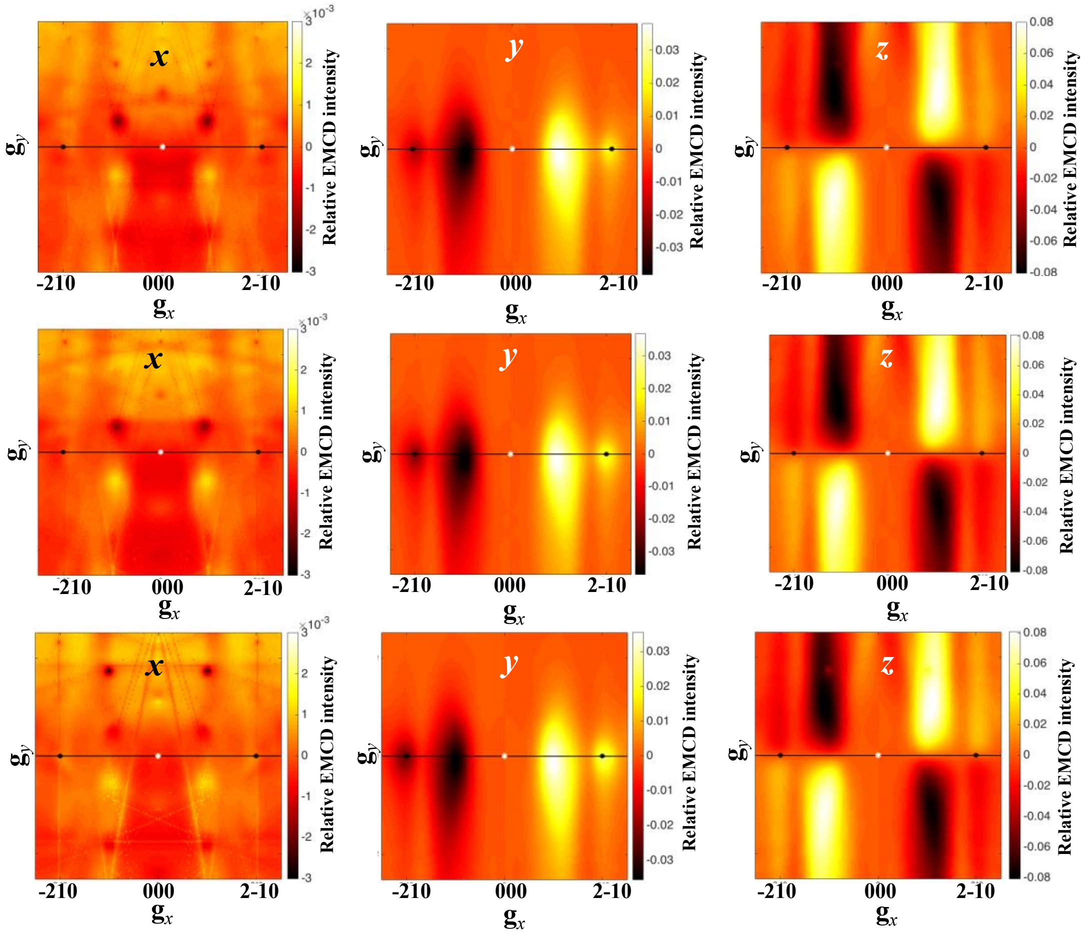

Supplementary Figure 8 | Effect of strain on the relative intensity of the EMCD signals for *hcp* Co under the (-210) three-beam case with the thickness of 20 nm and the acceleration voltage of 300 kV. The upper panel, middle panel and lower panel correspond to the strain of 3%, 0% and -3% in the (001) plane, respectively.
